# Hydrogen Bonding-Mediated Microphase Separation during the Formation of Mesoporous Novolac-Type Phenolic Resin Templated by the Triblock Copolymer, PEO-*b*-PPO-*b*-PEO

**DOI:** 10.3390/ma6115077

**Published:** 2013-11-07

**Authors:** Wei-Cheng Chu, Shih-Fan Chiang, Jheng-Guang Li, Shiao-Wei Kuo

**Affiliations:** Center for Nanoscience and Nanotechnology, Department of Materials and Optoelectronic Science, National Sun Yat-Sen University, Kaohsiung 804, Taiwan

**Keywords:** hydrogen bonding, microphase separation, triblock copolymer, mesoporous structure, phenolic resin

## Abstract

After blending the triblock copolymer, poly(ethylene oxide-*b*-propylene oxide-*b*-ethylene oxide) (PEO-*b*-PPO-*b*-PEO) with novolac-type phenolic resin, Fourier transform infrared spectroscopy revealed that the ether groups of the PEO block were stronger hydrogen bond acceptors for the OH groups of phenolic resin than were the ether groups of the PPO block. Thermal curing with hexamethylenetetramine as the curing agent resulted in the triblock copolymer being incorporated into the phenolic resin, forming a nanostructure through a mechanism involving reaction-induced microphase separation. Mild pyrolysis conditions led to the removal of the PEO-*b*-PPO-*b*-PEO triblock copolymer and formation of mesoporous phenolic resin. This approach provided a variety of composition-dependent nanostructures, including disordered wormlike, body-centered-cubic spherical and disorder micelles. The regular mesoporous novolac-type phenolic resin was formed only at a phenolic content of 40–60 wt %, the result of an intriguing balance of hydrogen bonding interactions among the phenolic resin and the PEO and PPO segments of the triblock copolymer.

## 1. Introduction

Mesoporous materials having controllable pore sizes are attractive to both the academic and industrial communities, because of their various applications in the fields of filtering, separating, sensing, catalysis and controlled drug release [1,2,3,4,5]. The self-assembly of amphiphilic block copolymers as templates has been used extensively in the structure-directed syntheses of many mesoporous materials [5,6]. In the bulk state, block copolymers self-assemble into various nanostructures, including lamellae, gyroids, hexagonally packed cylinders, spheres and more complicated structures [7,8,9,10]. Many large-pores, long-range-ordered mesoporous silicates have been prepared using amphiphilic block copolymers as templates [11,12]. In addition to inorganic/organic assembly of mesoporous silicas, organic/organic self-assembly has also been used to synthesize ordered mesoporous or nanoporous polymeric materials from, for example, phenolic resin [13,14], polyurethane (PU) [15], polystyrene (PS) [16] and polyethylene (PE) [17].

The self-assembly of amphiphilic block copolymers into different thermoset polymers (e.g., phenolic and epoxy resins) has been used widely to prepare long-range-ordered nanostructures and mesopores [18,19,20,21,22,23,24,25,26,27,28,29]. For example, Ikkala and Ruokolainen* et al.* prepared long-range-ordered mesoporous phenolic resins from the diblock copolymers poly(isoprene-*b*-2-vinylpyridine) (PI-*b*-P2VP) and poly(styrene-*b*-4-vinylpyridine) (PS-*b*-P4VP) as templates, with hexamethylenetetramine (HMTA) as the curing agent [30,31,32]. Zheng* et al.* also reported the formation of long-range-ordered nanostructures in phenolic thermosets after curing novolac and the diblock copolymer poly(styrene-*b*-ethylene oxide) (PS-*b*-PEO) with HMTA [33]. From previous studies, we have also reported the formation of long-range-ordered cylinder and gyroid nanostructures of mesoporous phenolic resin templated by poly(ethylene oxide-*b*-ε-caprolactone) (PEO-*b*-PCL) [11,12]. Long-range-ordered nanostructures can be formed after mixing uncured phenolic resins with block copolymers featuring a block that forms sufficiently strong hydrogen bonds with the phenolic OH groups, such that curing of the phenolic resin will preserve the self-assembled structure without undergoing macroscopic phase separation. In addition, controlled pyrolysis can be used subsequently to remove the structure-directing block copolymer template, leading to a monomodal mesoporous material featuring pores on a well-defined length scale [34,35,36,37,38]. The block copolymers used in those previous studies were, however, synthesized in the laboratory using complex and time-consuming techniques, such as anionic polymerization, atom transfer radical polymerization and ring opening polymerization [30,31,32,33,34,35,36,37,38]. Methods for modifying mesoporous structures employing commercial block copolymers would be simpler and more effective.

Recently, a family of ordered mesoporous carbon materials has been synthesized using poly(ethylene oxide)-*b*-poly(propylene oxide)-*b*-poly(ethylene oxide) (PEO-PPO-PEO) commercial triblock copolymers, such as F127 (EO_106_PO_70_EO_106_) and P123 (EO_20_PO_70_EO_20_), as templates [39,40,41,42]. Resol-type phenolic resin precursors have often been used as the carbon source. Ikkala* et al.* reported the blending of resol-type phenolic resins with PEO-PPO-PEO triblock copolymers of three different molecular weights, namely EO_8_PO_47_EO_8_, EO_17_PO_56_EO_17_ and EO_21_PO_47_EO_21_ [43]; they observed only disordered micelle structures in these phenolic/block copolymer blends. Novolac, another popular type of phenolic resin, has been difficult to use in the preparation of ordered mesoporous polymers and carbons. Because novolac has a linear chain and thermoplastic properties, it melts when heated at high temperatures and, therefore, must be cured with HMTA to avoid collapsing during the pyrolysis stage [12]; accordingly, such materials exhibit poor periodicity (mostly worm-like or disordered micelle mesoporous structures). Zhao* et al.* have prepared mesoporous carbons using F127 and P123 as templates [39,40,41,42]. To date, however, the preparation of regular, ordered mesoporous novolac phenolic resins templated by PEO-PPO-PEO has remained a challenge. The phenolic OH groups of novolac should interact with the ether groups of both the PEO and PPO block segments of PEO-PPO-PEO, making it miscible at relatively higher phenolic resin contents. To prepare a phenolic resin exhibiting long-range order, the OH groups of the phenolic should interact with only the ether groups of PEO and not with those of PPO [44]. The triblock architecture might allow anchoring of the potentially repulsive PPO domains, if hydrogen bonding occurs only with the PEO domains. As a result, the length of the PEO blocks should be larger than that of the PPO blocks to ensure that the OH groups of the phenolic resin interact primarily with the ether units of the PEO blocks. The repulsive PPO block might lead to a phenolic resin exhibiting a long-range-ordered, self-assembled nanostructure.

In this study, we prepared regular, ordered mesoporous phenolic resins templated by the triblock copolymer, F127 (EO_106_PO_70_EO_106_), because the PEO blocks were longer than those in the triblock copolymer, P123 (EO_20_PO_70_EO_20_). We had anticipated that the OH groups of the phenolic would form hydrogen bonds primarily with the ether groups of the PEO blocks at relative low phenolic resin content, but then would interact with the ether groups of the PPO block at relatively high phenolic resin content, possibly forming a variety of composition-dependent nanostructures (e.g., cylindrical, wormlike, spherical). Herein, we describe the phase behavior and hydrogen bonding interactions of phenolic nanostructures, which we investigated using differential scanning calorimetry (DSC), Fourier transform infrared (FTIR) spectroscopy, small-angle X-ray scattering (SAXS) and transmission electron microscopy (TEM).

## 2. Results and Discussion

### 2.1. Thermal Analyses of Phenolic Resin/Block Copolymer Blends

**Figure 1:**
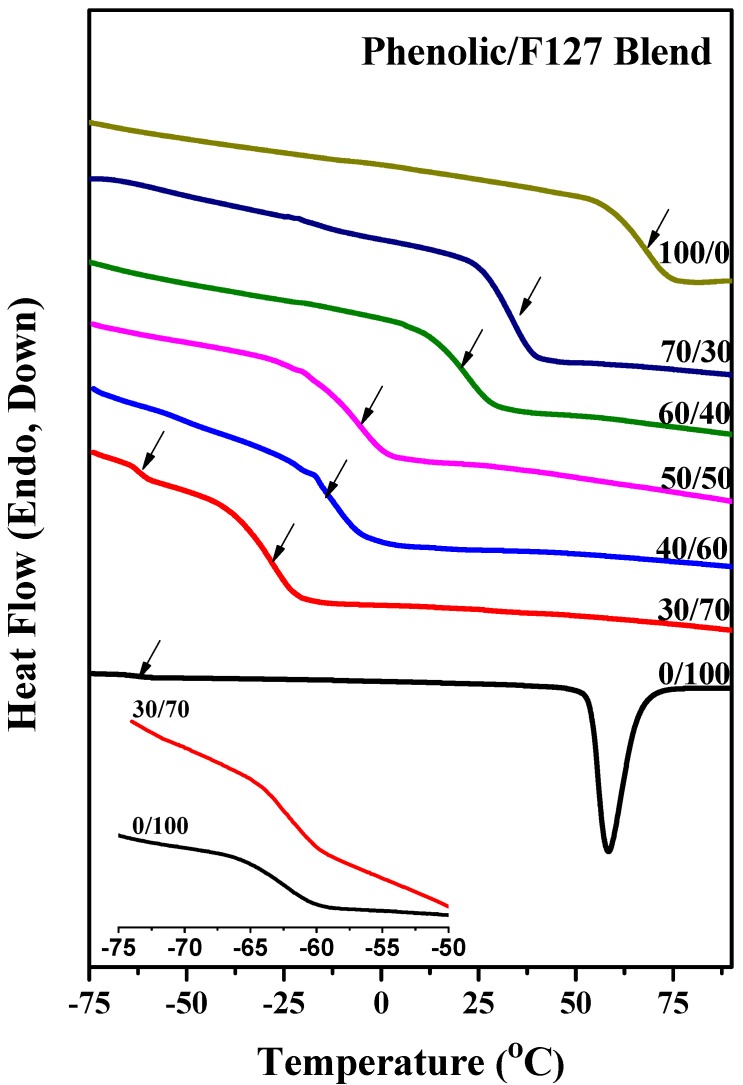
Differential scanning calorimetry (DSC) thermograms of phenolic/F127 blends at various compositions.

**Figure 2:**
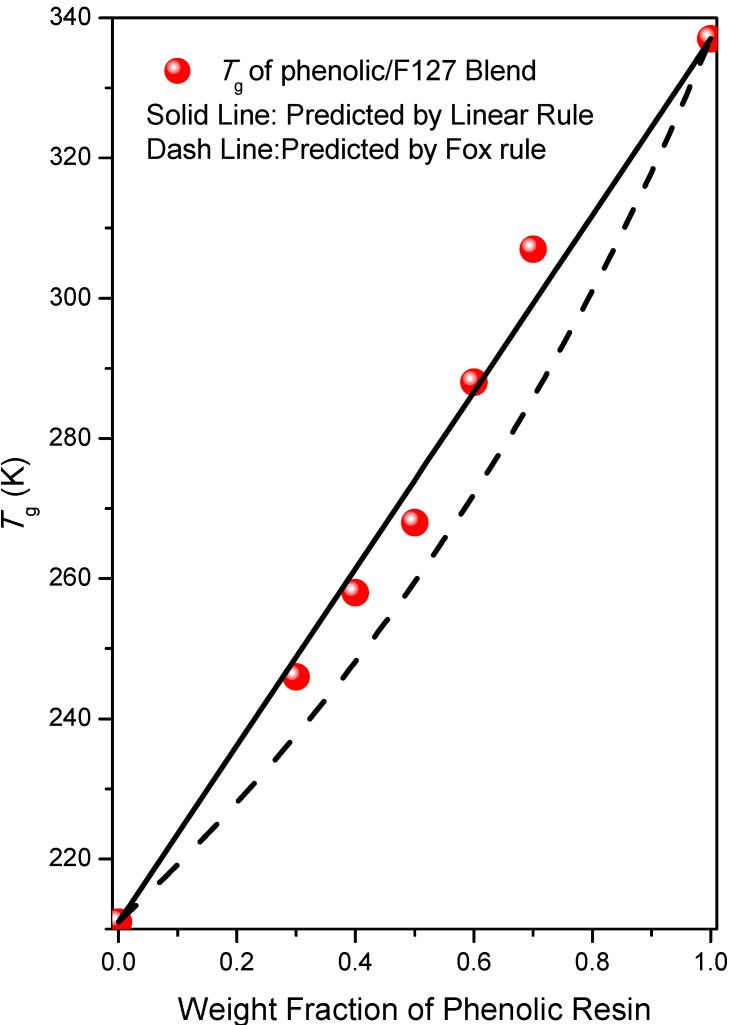
Glass transition (*T*_g_) behavior of phenolic/F127 blends at various compositions.

DSC is used extensively to investigate miscibility in polymer blends. The glass transition temperatures (*T*_g_) of the pure polymers used in this study, phenolic, PEO and PPO, were +64, −62, and −62 °C, respectively. Figure 1 presents conventional second-run DSC thermograms of phenolic/F127 blends of various compositions (not cured with HMTA) obtained at a heating rate of 20 °C/min. In the region from −60 to −75 °C, the pure F127 exhibited one glass transition at −62 °C, because the values of the *T*_g_ of PEO and PPO are very similar [45]. When blended with 30 wt % of phenolic resin, we observed two glass transitions at −62 and −27 °C, indicating that phase separation had occurred at this blend composition. The higher value of *T*_g_ (−27 °C) presumably arose from the phenolic/PEO phase formed through intermolecular hydrogen bonding between the OH groups of the phenolic resin and the ether groups of PEO; the lower value (−62 °C) presumably arose from the PPO block segment. We would expect the inter-association equilibrium constant for phenolic/PEO to be larger than that for phenolic/PPO, based on extensive previous studies of the hydrogen bonding interactions of PEO and PPO [44,46,47,48]. Further increasing the phenolic resin content, the two glass transitions became a single one, with a shift toward higher temperature. A single value of *T*_g_ strongly suggests that these blends are fully miscible with a homogeneous amorphous phase. At relatively high contents of phenolic resin, the OH groups of the phenolic resin could interact with the ether groups of both the PEO and PPO block segments, thereby inducing total miscibility for the blend (disordered structure). Figure 2 reveals that a linear rule can be used to predict of the single values of *T*_g_ for the phenolic/F127 blend; these values are higher than those predicted using the Fox rule. This behavior is similar to that of the many ternary blends that become totally miscible when the content of a polymer presenting OH group is relatively high [49,50].

### 2.2. FTIR Spectroscopic Analyses of Phenolic Resin/Block Copolymer Blends

**Figure 3:**
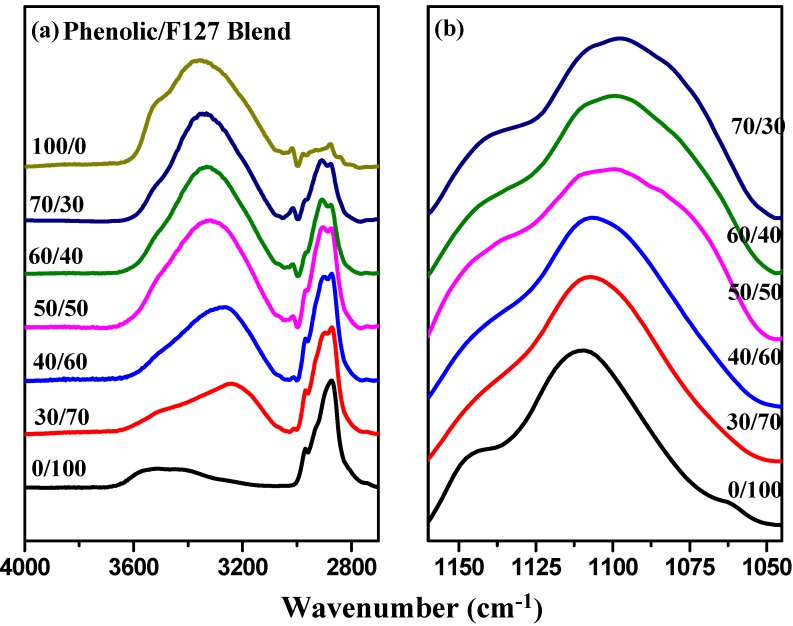
FTIR spectra, recorded at room temperature, of phenolic/F127 blends at various compositions: (**a**) O–H and (**b**) C–O–C stretching regions.

Infrared spectroscopy is a highly effective method for investigating hydrogen bonding interactions in polymer blend systems. Here, we used FTIR spectroscopy to provide evidence for hydrogen bonding within the phenolic/F127 blend. The signals in the OH stretching region of the room-temperature FTIR spectra for the blends of the F127 triblock and phenolic, cast from tetrahydrofuran solutions, were sensitive to the presence of hydrogen bonds (Figure 3a). The spectrum of phenolic resin featured two major unresolved bands in the OH stretching region, corresponding to the free OH groups at 3525 cm^−1^ and a broad band centered at 3360 cm^−1^ arising from the absorption of hydrogen-bonded OH groups (self-association). The intensity of the signal for the free OH groups decreased gradually upon increasing the content of the F127 triblock copolymer, as we had expected. The broad band of the phenolic resin at 3360 cm^−1^ became even broader when it was blended with F127 at higher phenolic contents, indicating that the OH groups of the phenolic interacted with the ether groups of both the PEO and PPO blocks. At lower phenolic contents, the signal for the OH groups involved in [OH**···**ether] hydrogen bonds with the PEO block appeared at a lower wavenumber of 3275 cm^−1^ [48], suggesting that the average hydrogen bonding strength followed the order [OH**···**ether] > [OH**···**OH], consistent with the values of *T*_g_ being higher than those predicted using the Fox rule.

Figure 3b displays the ether C–O–C stretching region of the phenolic/F127 blends at room temperature. According to a previous study, the free ether units of PPO and PEO provide bands near 1112 and 1104 cm^−1^, respectively; in addition, the signals for the ether groups of PPO and PEO hydrogen bonded to OH groups appear at 1090 and 1082 cm^−1^, respectively [44]. In this study, the signal for the ether units in the triblock copolymer, F127, was located at 1109 cm^−1^, corresponding to a combination of the free ether moieties of the PPO and PEO blocks. This peak shifted to a lower wavenumber upon increasing the content of phenolic resin. When an ether forms a hydrogen bond with a phenolic resin, the lone pair of electrons on the oxygen atom are withdrawn by the OH group of the phenolic, thereby decreasing the electron density from the ether C–O bonds and inducing a shift in the band to a lower wavenumber. The main ether peak at 1109 cm^−1 ^shifted to 1106 cm^−1 ^at a phenolic resin content of 30 and 40 wt %, to 1100 cm^−1 ^at 50 wt % and to 1098 cm^−1 ^at 60 and 70 wt %. In addition, a shoulder appeared at 1074 cm^−1 ^at relatively higher phenolic resin contents; this signal corresponded to the ether units of the PPO block hydrogen bonded with the phenolic resin.

### 2.3. Thermal Analyses of Phenolic Resin/Block Copolymer Blends with HMTA Curing

**Figure 4:**
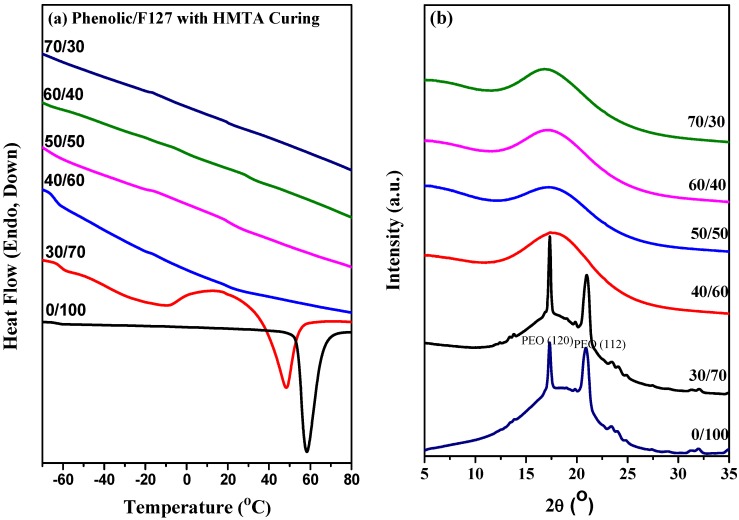
(**a**) DSC thermograms and (**b**) wide-angle X-ray diffraction analyses of phenolic/F127 blends with hexamethylenetetramine (HMTA) curing. PEO, poly(ethylene oxide).

Next, we investigated the thermal behavior of the phenolic/F127 blends prepared with HMTA as a curing agent. Prior to thermal curing, all of our binary mixtures of the phenolic and F127 were homogenous and transparent. After thermal curing, the phenolic resin remained homogenous and transparent, but became dark red in color, suggesting that macrophase separation had not occurred. Nevertheless, we could not explain reaction-induced microphase separation merely in terms of sample clarity. Figure 4a presents conventional second-run DSC thermograms of phenolic/F127 blends with HMTA at various compositions, recorded at a heating rate of 20 °C/min. The blends exhibited a melting temperature that corresponded to that of crystalline PEO. Meanwhile, both the pure F127 and phenolic/F127 = 30/70 with HMTA curing exhibited two strong diffraction peaks at 17.29 and 21.04, arising from the crystal structure of the PEO blocks (Figure 4b) [36]. Prior to thermal curing, the phenolic/F127 blend featured the amorphous phase of PEO and PPO at a phenolic resin content of 30 wt %; it became a crystalline phase after thermal curing with HMTA, due to a mechanism based on reaction-induced phase separation, which is caused by the increase in the average molar mass of the polymer superimposed by possible variations of the interaction parameter with thermal curing. Further increasing the content of phenolic resin caused all of the systems to become amorphous, without melting temperature (DSC), consistent with the wide-angle X-ray diffraction analyses. Furthermore, the glass transition of the PPO block segment was observed at −62 °C for phenolic/F127 = 40/60 with HMTA thermal curing; this signal was not apparent in the absence of HMTA thermal curing. Clearly, microphase separation occurred in the cured blends of the phenolic thermosets with F127, possibly through reaction-induced demixing from homogenous mixtures.

**Figure 5:**
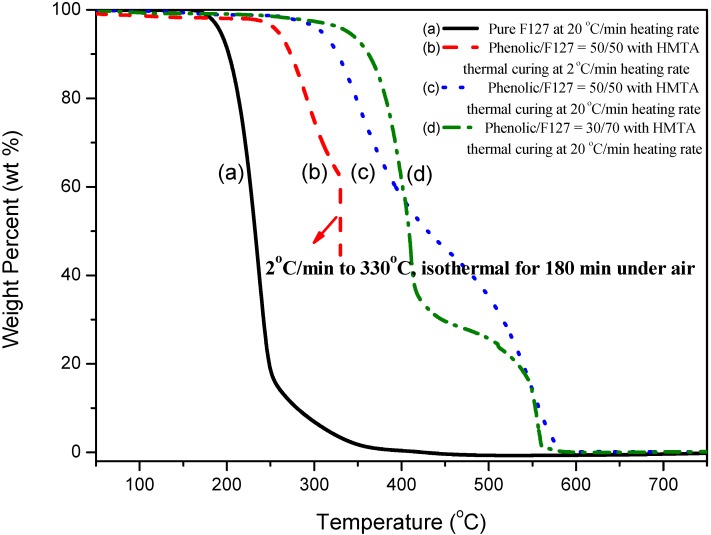
TGA analyses of (**a**) the pure triblock copolymer, F127, at a heating rate of 20 °C/min; (**b**) phenolic/F127 = 50/50 with HMTA thermal curing at a heating rate of 2 °C/min and after isothermal treatment for 3 h; (**c**) phenolic/F127 = 50/50 with HMTA thermal curing at a heating rate of 20 °C/min; and (**d**) phenolic/F127 = 30/70 with HMTA thermal curing at a heating rate of 20 °C/min.

Figure 5 displays thermogravimetric analyses, under an atmosphere of air, of the phenolic/F127 blends with HMTA thermal curing. Pure F127 underwent its main degradation at a temperature near 230 °C (Figure 5a). At a phenolic content of 50 wt %, this thermal degradation temperature increased to 350 °C at a heating rate of 20 °C/min (Figure 5c), and at a phenolic content of 30 wt %, this thermal degradation temperature also increased to 380 °C at a heating rate of 20 °C/min (Figure 5d). If we selected a heating rate of 2 °C/min, however, the F127 readily decomposed at 330 °C, as it did after isothermal treatment for 3 h at 330 °C (Figure 5b). This result implied that the F127 template could be removed readily through pyrolysis in air, leaving behind the phenolic resin.

### 2.4. SAXS, TEM and BET Analyses of Mesoporous Phenolic Resins

**Figure 6:**
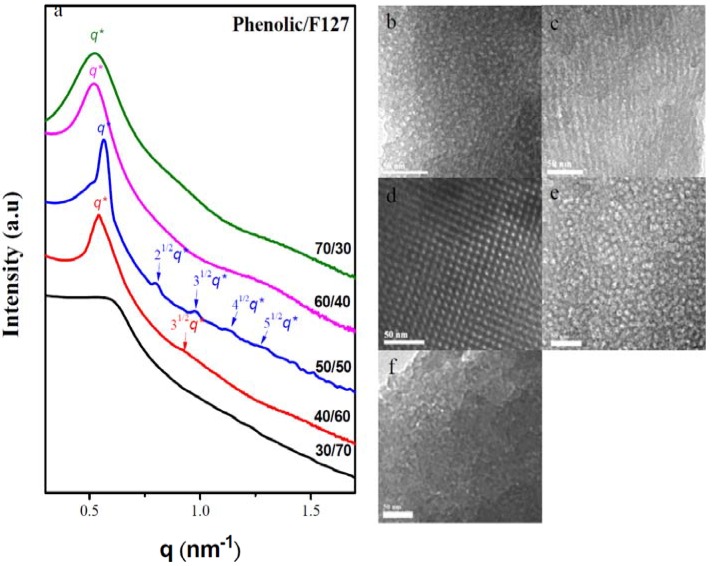
(**a**) Small-angle X-ray scattering (SAXS) patterns and (**b**–**f**) transmission electron microscopy images of mesoporous phenolic templated by F127 at phenolic/F127 ratios of (**b**) 30/70; (**c**) 40/60; (**d**) 50/50; (**e**) 60/40 and (**f**) 70/30.

We recorded SAXS profiles of the mesoporous phenolic resins templated by the triblock copolymer, F127, at room temperature to confirm their self-organized mesoporous morphologies. Figure 6 reveals that the phenolic content influenced the formation of the mesostructures. In the SAXS profiles of the mesoporous phenolic resins templated by F127 (Figure 6a), we observe a broad peak corresponding to the disordered mesoporous phenolic resin formed at a relatively low phenolic content (30 wt %), consistent with the TEM image in Figure 6b. The blend prepared with a phenolic content of 40 wt % provided a single broad peak at a value of q* of approximately 0.54 nm^−1^ (*d* = 11.62 nm) with second-order reflections at 3^1/2^q* (see inset) that correspond to the short-range order of a hexagonally packed cylinder structure; this result is consistent with the TEM image in Figure 6c, which displays a worm-like structure. The SAXS pattern of the blend with 50 wt % phenolic resin exhibits a strong and narrow scattering peak at a value of *q* of 0.56 nm^−1^ (*d* = 11.21 nm). The higher-order peaks are clearly evident at 2^1/2^:3^1/2^:4^1/2^:5^1/2^q*, corresponding to a body-centered-cubic spherical structure, consistent with the TEM image in Figure 6d. When the phenolic content was 60 wt %, the SAXS pattern featured only a single broad peak at a value of q* of approximately 0.52 nm^−1^ (*d* = 12.07 nm), due to incomplete disordering of the mesoporous micellar structure at this composition; the TEM image in Figure 6e reveals a disordered mesoporous micellar structure for this sample, similar to that previously reported for a phenolic/PS-*b*-P4VP = 70/30 system [30]. Further increasing the phenolic resin content to 70 wt % resulted in a disordered structure, based on the appearance of broad peaks in the SAXS pattern, indicating the absence of long-range order in the structure; the TEM image confirmed this result (Figure 6f).

**Scheme 1:**
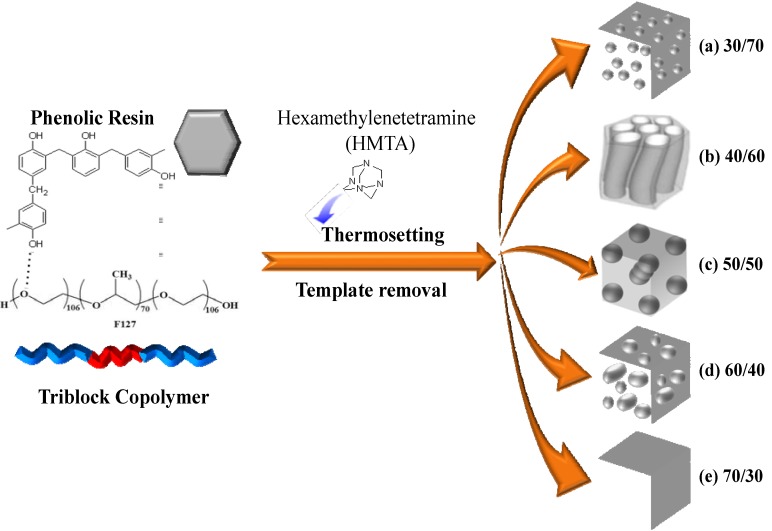
Morphologies of mesoporous phenolic resins templated by F127 at various compositions.

**Figure 7:**
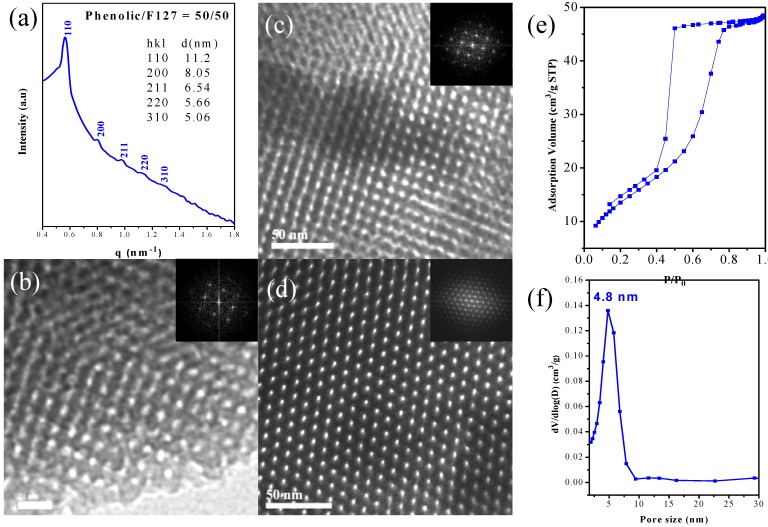
(**a**) SAXS pattern; (**b**–**d**) TEM images viewed from (**b**) (110); (**c**) (100) and (**d**) (111) (insets: corresponding Fast Fourier transform); (**e**) N_2_ adsorption/desorption isotherm and (**f**) pore size distribution curve of the *bcc* mesoporous phenolic templated by F127 at a phenolic resin content of 50 wt %.

Therefore, the key factor for preparing mesoporous phenolic structures templated by the triblock copolymer, F127, is that the phenolic OH groups should form hydrogen bonds with the ether units of the PPO block, but the fraction of hydrogen-bonded PPO ether moieties should not be so high that a miscible disorder system forms. As a result, the regular mesoporous phenolic resin that forms, templated by the triblock copolymer, F127, is strongly dependent on the phenolic resin content. Scheme 1 summarizes the mechanism of the formation of mesoporous phenolic resins templated by the triblock copolymer, F127. At lower phenolic contents (<30 wt % phenolic), the system contained insufficient phenolic resin relative to the template F127, forming a disordered structure (Scheme 1a). At higher phenolic content (>70 wt % phenolic; Scheme 1e), a completely miscible disordered structure formed, because the phenolic OH groups formed hydrogen bonds with both the PEO and PPO blocks, according to FTIR spectroscopy. As a result, the self-organized mesoporous phenolic resin formed only at phenolic contents of 40–60 wt %, through an intriguing balance of the interactions of phenolic, PEO and PPO (Scheme 1b–d). Most importantly, we have developed a simple evaporation-induced self-assembly (EISA) method for the synthesis of new mesoporous phenolic resin having a *bcc* structure when applying the copolymer, F127, having a large volume fraction of its EO block segments, as the template at phenolic/F127 = 50/50 (Scheme 1d). Figure 7 summarized the data from the SAXS, TEM and Brunauer-Emmett-Teller (BET) analyses. The SAXS pattern of this bcc-type mesoporous phenolic resin exhibited (Figure 7a) a strong reflection with a large *d*-spacing of 11.21 nm and another four strong reflections at values of *q* of 0.79, 0.97, 1.12 and 1.25 nm^−1^; this SAXS pattern could be indexed as having (110), (200), (211), (220) and (310) reflections, corresponding to a cubic structure (*Im *3 *m* space group). In addition, we confirmed the structural ordering and cubic symmetry of this material through TEM analyses.

Figure 7b–d display TEM images of the bcc-type mesoporous phenolic resin with different orientations ((100), (110) and (111) planes, respectively), consistent with a three-dimensional (3D) cubic cage structure having a large *d*-spacing—a material having potential advantages when applied in catalysis and separation. Notably, these synthesis conditions enabled the facile and highly reproducible preparation of a large-cage 3D cubic mesoporous phenolic resin having *Im *3 *m* symmetry. We obtained further information regarding the textural properties of the materials from N_2_ adsorption/desorption isotherms measured at 77 K. Figure 7e presents the N_2_ sorption isotherms of the cubic mesoporous phenolic resin sample. The sample displays individual type-IV isotherms, exhibiting an apparent H_2_ hysteresis loop characteristic of a cage-like mesoporous material. A sharp capillary condensation step appeared for this sample, suggesting uniform pore dimensions and high-quality ordering of the materials, in agreement with the TEM and SAXS data. Pore size distribution analysis revealed (Figure 7f) a well-ordered cubic structure having pores with an average diameter of approximately 4.8 nm. The significant width of the hysteresis loops also observed for the Santa-Barbara-16 (SBA-16) and Fudan University-16 (FDU-16) samples studied herein suggested cage-like pore shapes [51,52].

**Figure 8:**
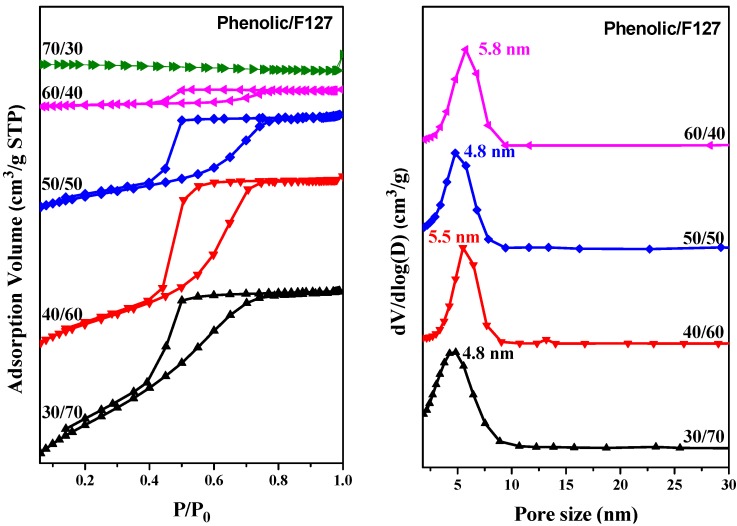
(**a**) N_2_ adsorption/desorption isotherms and (**b**) pore size distribution curves of mesoporous phenolic templated by F127 at phenolic/F127 weight fractions of 30/70, 40/60, 50/50, 60/40 and 70/30.

Figure 8a summarizes the N_2_-sorption phys-isotherm of other mesoporous phenolic resins templated by the triblock copolymer, F127. The mesoporous phenolic samples templated by F127 triblock copolymers at phenolic contents of 30, 40, 50 and 60 wt % exhibited representative type-IV curves. We did not, however, find such a curve at a phenolic content of 70 wt %, indicating that no mesoporous structure formed at this composition. The hysteresis loops gradually changed from H_1_-type loops at phenolic contents of 30 and 40 wt % to an H_2_-type loop at a phenolic content of 50 wt %. Because an H_1_-like hysteresis loop is usually attributed to uniformly branched and cylindrical pores, such a change in the feature of the hysteresis loops may reflect a change in the mesoporous structure from a cylinder to *bcc* structure. Based on the model of Harkins and Jura [53], the mean pore sizes measured from the adsorption branch for mesoporous phenolic resins prepared at phenolic contents of 30, 40, 50 and 60 wt % were 4.8, 5.5, 4.8 and 5.8 nm, respectively. Table 1 summarizes the BET surface areas, pore volumes and Barrett-Joyner-Halenda BJH pore sizes of our mesoporous phenolic materials. The total BET surface area and the total pore volume decreased upon increasing the phenolic content of the templating triblock copolymer, F127. The low BET surface areas in this study may come presumably from partial collapse of the well-defined structure during pyrolysis. To the best of our knowledge, this study is the first in which a long-range-ordered, bcc-type, mesoporous, novolac-type phenolic resin has been fabricated using an EISA strategy.

**Table 1:** Textural properties of mesoporous phenolic resins templated by F127.

Sample Phenolic/F127	*d* (nm) ^a^	Pore Size (nm)	*S*_BET_ (m^2^/g) ^b^	Pore Volume (cm^3^/g)
30/70	10.6	4.8	155	0.15
40/60	11.6	5.5	82	0.11
50/50	11.2	4.8	52	0.08
60/40	12.1	5.8	10	0.01
70/30	12.1	–	–	–

^a^ The *d*-spacing was calculated from the first SAXS peak, using the formula *d* = 2π/q*; ^b^ Total Brunauer-Emmett-Teller (BET) surface area.

## 3. Experimental Section

### 3.1. Materials

The triblock copolymer, Pluronic F127 (EO_106_PO_70_EO_106_;* M*_n_ = 12,600), was purchased from Aldrich (St. Louis, MO, USA). The phenolic resin was synthesized through a condensation reaction mediated by sulfuric acid, resulting in an average molecular weight (*M*_n_ = 500) similar to those described previously [54,55].

### 3.2. Phenolic/F127 Blends

Phenolic resin and Pluronic F127 were dissolved in THF and stirred for 30 min to form a homogeneous solution. The sample was poured onto an aluminum plate, and then, the THF was slowly evaporated at room temperature for 3 days; the sample was subsequently vacuum-dried at 30 °C for 24 h.

### 3.3. Mesoporous Phenolic Resins

Phenolic resin, HMTA and Pluronic F127 were dissolved in THF and stirred for 30 min to form a homogeneous solution. The sample was poured onto an aluminum plate, and then, the THF was slowly evaporated at room temperature for 3 days; the sample was subsequently vacuum-dried at 30 °C for 24 h. Curing of the sample was performed using the following temperature profile: 100 °C for 2 h, 150 °C for 2 h and 190 °C for 2 h. Pyrolysis of the cross-linked sample was performed by calcinating at 330 °C for 3 h in the absence of a protective gas atmosphere. Calcination was conducted in a furnace operated at a heating rate of 2 °C/min.

### 3.4. Characterization

Thermal analysis was performed using a Q-20 differential scanning calorimeter (TA Instruments Q-20, New Castle, DE, USA) operated at a heating rate of 20 °C and a cooling rate of 5 °C/min from +150 to −90 °C under N_2_; the sample weighed between 5 and 10 mg. The thermal stability of the samples was characterized using a TA Q-50 Thermogravimetric Analyzer TA Instruments (New Castle, DE, USA) operated under N_2_ or air; the sample (*ca*. 7 mg) was placed in a Pt cell and heated at a rate of 20 °C /min from 30 to 800 °C at a N_2_ flow rate of 60 mL/min or at 1.4 °C/min to 330 °C and then held isothermally for 180 min at an air flow rate of 60 mL/min. FTIR spectra of the samples were recorded with a Nicolet Avatar 320 FTIR spectrometer (Thermo Scientific, Waltham, MA, USA) and the conventional KBr disk method. SAXS experiments were performed using the small/wide angle X-ray scattering (SWAXS) instrument at the BL23A1 beamline of the National Synchrotron Radiation Research Center, (NSRRC), Taiwan; the X-ray beam had a diameter of 0.5 mm and a wavelength (λ) of 1.24 Å; the *d*-spacings were calculated using the formula *d* = 2π/*q*, where *q* is the scattering vector. TEM images were recorded using a JEOL 2100 microscope (Tokyo, Japan) operated at 200 kV; samples for TEM measurement were suspended in ethanol and supported onto a holey carbon film on a Cu grid. Nitrogen adsorption/desorption isotherms were measured at −196 °C using an accelerated surface area and porosimetry system (ASAP) 2020 analyzer (Micromeritics, GA, USA); prior to measurements, the samples were degassed under vacuum at 200 °C for at least 6 h. The Brunauer-Emmett-Teller (BET) method was employed to calculate the specific surface areas; using the Broekhoff-de Boer (BdB) sphere model, the pore volumes and pore size distributions were derived from the adsorption branches of the isotherms; the total pore volumes were estimated from the amounts adsorbed at a relative pressure (*P*/*P*_0_) of 0.995.

## 4. Conclusions

We have employed DSC, TEM, SAXS and FTIR spectroscopy to investigate the miscibility, phase behavior and hydrogen bonding within phenolic/F127 triblock copolymer blends. DSC and FTIR spectroscopy provided evidence for the ether units of the PEO and PPO blocks being hydrogen bond acceptors for the OH groups of the phenolic resin. SAXS and TEM analyses indicated that phenolic/F127 blends of different compositions resulted in different microphase-separated structures, mediated through hydrogen bonding interactions. Most importantly, we report the first examples of mesoporous novolac-type phenolic resins formed after mild pyrolysis of the templating triblock copolymer, F127. The mesoporous phenolic resins exhibited a long-range-ordered *bcc* structure only when the phenolic content was 50 wt %, formed through a mechanism involving an intriguing balance of interactions among the phenolic and the components of the block copolymer.
